# Freiburg Questionnaire of linguistic pragmatics (FQLP): psychometric properties based on a psychiatric sample

**DOI:** 10.1186/s12888-014-0374-9

**Published:** 2014-12-24

**Authors:** Andreas Riedel, Heejung Suh, Verena Haser, Ismene Hermann, Dieter Ebert, Dieter Riemann, Emanuel Bubl, Ludger Tebartz van Elst, Lars P Hölzel

**Affiliations:** Clinic for Psychiatry and Psychotherapy, University Medical Center Freiburg, Hauptstr. 5, Freiburg, 79104 Germany; Department of English Linguistics, University of Freiburg, Freiburg, Germany

**Keywords:** Autism, Asperger’s syndrome, Questionnaire, Linguistic pragmatics, Screening, Clinical diagnostics, Language comprehension

## Abstract

**Background:**

Asperger’s Syndrome (AS) is an autism spectrum disorder that is characterized by significant difficulties in social interaction and nonverbal communication, and restricted and repetitive patterns of behavior and interests. Difficulties with respect to pragmatic speech, reading emotional and social cues, differentiating between fact and fiction, and taking into account the influence of context on a statement are commonly described features. However, hitherto established questionnaires did not focus on these symptoms.

**Methods:**

In this study we present a short (11 questions) questionnaire which focuses on self-rated pragmatic speech abilities, the Freiburg Questionnaire of linguistic pragmatics (FQLP). Psychometric properties of the questionnaire were explored in a sample of 57 patients with Asperger’s Syndrome, 66 patients with other psychiatric disorders, and a convenience sample of 56 people.

**Results:**

Reliability analysis showed a high Cronbach’s α. Strong correlations could be demonstrated for the FQLP with the Autism Quotient and the Empathy Quotient. Concerning divergent validity a moderate correlation was found between the FQLP and self-rated symptoms of personality disorders. No significant correlation was found between the FQLP and the vocabulary skills. The receiver operating characteristics curve showed an excellent diagnostic accuracy of the FQLP (.97).

**Conclusions:**

As the control group consisted of people without mental disorder and patients with different psychiatric disorders, the results indicate that the construct examined by the FQLP is quite specific to the peculiarities of AS. The FQLP is a reliable, brief and valid instrument. First results regarding sensitivity and specificity are highly promising.

**Electronic supplementary material:**

The online version of this article (doi:10.1186/s12888-014-0374-9) contains supplementary material, which is available to authorized users.

## Background

Asperger’s Syndrome (AS) is an autism spectrum disorder (ASD) that is characterized by significant difficulties in social interaction and nonverbal communication, and restricted and repetitive patterns of behavior and interests. According to the criteria of the ICD-10 (F84.5, [1]) AS is distinguished from early childhood autism by unimpaired speech and cognitive development. A closer look shows, however, that patients with AS have peculiarities in their understanding and use of language, well characterized by Hans Asperger [2]. In his original publication, he pointed out that while children with AS show very good abilities regarding the terminology of language and formulate correctly and precisely, they do have difficulties with respect to nonverbal communication, reading emotional and social cues in and “between” the lines of the speaker, differentiating between fact and fiction, taking into account the influence of context on a statement, and modifying communication depending on priorities [2]. In recent years, many studies have shown that people with ASD and AS show abnormalities in the pragmatic understanding and the use of language [3-8]. The scientific results overall are inconsistent, however, and explanations of these peculiarities have remained controversial until now [9]. Many of the described characteristics refer to an inability to respond adequately in a specific context. This is already apparent in children who often cannot select deictic expressions (*I, you*) correctly [10]. Grownups often show difficulties comprehending sentences with several meanings since they do not take the context into account sufficiently [3]. Problems in understanding metaphors illustrate that comprehending non-literal language also seems to pose a difficulty [11]. Peculiarities were also found in the understanding of figures of speech [12] and irony [13]. Shortcomings in the understanding of indirect language have also been shown [14]. Interpreting utterances in their social context seems to pose particular difficulties to patients with AS. They often misuse forms of politeness and are less able to narrate coherently and clearly than people without AS [15]. While the majority of authors assume a specific problem of pragmatic and/or figurative language in autism, this hypothesis is still matter of debate: Some authors argue that most of the findings mentioned above are just an artifact of a problem of general language comprehension, which was not controlled for [16,17].

A preliminary study [18] showed, however, that people with AS are aware of differences in the way they comprehend language (irrespective of whether or not these differences are genuinely pragmatic in nature). Based on these results we assume that the self-perception of language abnormalities could be an important aspect in the assessment of AS. To date, no validated screening questionnaire for AS focusing on the self-perception of language comprehension exists. Hitherto established self-report questionnaires concern social understanding (Autism Quotient, AQ, [19]), repetitive behavior (AQ), empathy (Empathy Quotient, EQ, [20]) and systemized thinking (Systemizing Quotient, SQ, [21]). In the Social Communication Questionnaire and the Social Responsiveness Scale [22-24] several language related symptoms are addressed. However, there is no focus on the self-perception of the language comprehension. This study aims to fill this gap.

## Methods

This study aims to investigate psychometric properties of the Freiburg Questionnaire of linguistic pragmatics (FQLP). Furthermore, we want to examine whether this questionnaire might be a useful tool in the diagnostic work up of autism spectrum disorders based on a group of diagnosed patients with AS, a convenience sample and a group of patients with other psychiatric diagnoses. Our aim is not to show whether the FQLP can serve as the sole tool to diagnose AS. Rather, our concern is to assess to what extent it can serve as a useful additional tool in the diagnostic process and as a screening instrument for AS.

### Participants

The aim was to include at least 50 patients with Asperger’s Syndrome (AS-group), a convenience sample (C-group) of at least 50 persons, and at least 50 patients with other psychiatric disorders (depression, schizophrenia, attention-deficit-hyperactivity-disorder, obsessive-compulsive disorder, borderline personality disorder: PD-group) in our study. The AS-group was recruited in our outpatient-clinic, the C-group was recruited using personal contacts of the authors, and patients of the PD-group were either acute or former patients of our clinic. They were recruited on clinical wards, from our outpatient-clinic, or contacted by mail. Upon agreement, the questionnaires were sent out by mail and the participants were requested to complete them. All subjects gave written informed consent before they participated in the study.

Organic brain disease, mental retardation and developmental disorders – apart from AS – served as exclusion criteria. In cases of doubt, MRTs, EEGs, tests of word pool (Multiple choice Vocabulary Test (MWT-B) [25]), intelligence tests (HaWIE [26], Raven’s Standard Progressive Matrices [27]), human genetic examinations and further clinical examinations were carried out. When indications of organic brain diseases, mental retardation, or other developmental disorders arose as a result, the patients concerned were excluded. All participants were native German speakers.

### Instruments

The FQLP is a self-report questionnaire which was developed in a previous project of our group [18]. Its questions are directed towards different pragmatic aspects of language comprehension. The FQLP was presented to the participants in German. An English version of the questionnaire was translated using the “forward-backward-method” [28]. Two items (06 and 12) of the original version were excluded later (see below); in the Additional files 1 and 2 we provide the FQLP in English and in German with 11 items.

The following 13 questions were to be answered according to these categories:

- *I agree / I tend to agree / I tend not to agree / I do not agree*
- 01: My comprehension of language differs from that of other people
- 02: I often don’t understand what other people are saying to me
- 03: In conversation, I find metaphors and/or sayings irritating
- 04: I intuitively comprehend metaphors and/or sayings I have never heard before
- 05: I consider metaphors and/or sayings to be unnecessary
- 06: Metaphors and/or sayings create visual images in my mind
- 07: I recognise expressions which are not meant literally because I have heard them in past and I misunderstood them then
- 08: I usually recognise irony easily
- 09: At school, I often misunderstood what my teachers and classmates said to me
- 10: I have made a conscious effort to improve my comprehension of metaphors/sayings
- 11: I use rational analysis to work out the meanings of metaphors etc.
- 12: To me, a sentence is the sum of its words
- 13: In an ideal language, there would be no ambiguity of meaning

For every *I agree*, the participants were given one point; for every I *tend to agree,* two points*.* For every *I tend not to agree,* three points and for every *I do not agree* four points*.* Items 4 and 8 were inversely scored.

In addition to the FQLP, participants were asked to complete the Autism questionnaire [19] and the Empathy questionnaire [20], as well as the Multiple choice Vocabulary Test (MWT-B) by Lehrl [25] in order to assess their linguistic abilities and vocabulary. In addition, the Symptom Checklist SCL-K-9 [29] and the Standardized Assessment of Personality - Abbreviated Scale (SAPAS) [30] were presented in order to assess general psychiatric symptoms and personality traits. Further, the following demographic information was captured by questionnaire: age, sex, familial status, relationship status, educational status, professional qualification and current medical diagnosis.

### Psychometric analysis

In order to clarify the factorial structure of the questionnaire an explorative factor analysis was conducted by means of a principal axis factor analysis (PAF) [31]. The number of factors to be extracted was determined under observance of the Kaiser-Guttman criterion [32] and the Scree-Test [33]. Second, reliability analysis was performed including discrimination (corrected item-total correlation), difficulty (mean) for each item and internal consistency (Cronbach’s α) for the whole scale. Third, construct validity was investigated by analysing convergent and divergent validity with respect to related constructs. We investigated the correlation of the self-rated pragmatic speech abilities (FQLP) with the self-rated symptoms of AS (AQ, EQ), self-rated symptoms of personality disorders (SAPAS), self-rated psychological distress (SCL-9), education and word pool (MWT-B). Pragmatic speech abilities perceived as low were hypothesized to be specific to patients with AS. Therefore, in patients with AS, the self-rated pragmatic speech abilities (FQLP) were supposed to be closely associated with self-rated symptoms of AS (AQ and EQ). We predicted pragmatic speech ability to be distinguishable from general psychopathology. However, as AS in adulthood has a high rate of comorbidities [34,35], we did not assume pragmatic speech abilities to be totally independent of ratings of personality disorders or psychological distress. Given this high comorbidity, we hypothesized the self-rated pragmatic speech abilities to be moderately associated with symptom ratings of personality disorders (SAPAS) or psychological distress (SCL-K-9). Furthermore, we predicted that people with AS have normal speech abilities and word pool with isolated deficits in pragmatic speech. Thus, validity was additionally examined by correlating pragmatic speech abilities (FQLP) with vocabulary (MWT-B). Although both measure speech-related constructs, we predicted self-rated pragmatic speech abilities (FQLP) to correlate only moderately with word pool (MWT-B). Fourth, criterion validity was explored by comparing the self-rated speech abilities (FQLP) of the AS-sample, the convenience sample and the psychiatric sample. The criterion validity was examined by comparing mean differences between the AS-group, the PD-group and the C-group. We controlled for differences in gender, age and education statistically by including them in the generalized linear model. Fifth, the diagnostic accuracy of the FQLP in identifying patients with AS was assessed through receiver operating characteristic analysis [36]. Sensitivity and specificity ratios were calculated.

In the case of missing item data (only scales), up to 30% were replaced using the expectation-maximization algorithm (EM).

SPSS (IBM SPSS Statistics 20.0.0) was used for all statistical analyses.

### Clinical diagnostics

Since the results of this analysis are highly dependent on the validity of the diagnosis given, we will describe in detail how the diagnosis of AS was established: At the Clinic of Psychiatry and Psychotherapy, University Medical Center Freiburg, there is a long established center for the assessment and treatment of children, adolescents and adults with autism spectrum disorder (university center for autism spectrum Freiburg, UZAS; http://www.uniklinik-freiburg.de/psych/live/patientenversorgung/schwerpunkte/schwerpunkt-asperger/ziele.html). The clinical diagnosis of autism spectrum disorders and AS is established as a consensus diagnosis of a multiprofessional team following the recommendations of the NICE guidelines (National Institute for Health and Clinical Excellence: Autism in Adults: full guideline DRAFT (December 2011; http://www.nice.org.uk/guidance/conditions-and-diseases/mental-health-and-behavioural-conditions/autism). According to these guidelines “a number of key components […] should form the basis of any comprehensive assessment of an adult with possible autism, as follows: the core symptoms of autism include social interaction, communication and stereotypical behavior; a developmental history spanning childhood, adolescence and adult life; the impact on current functioning including personal and social functioning, educational attainment and employment” (NICE 2012 page 134/135). At the UZAS the diagnostic principles are realized in a structured way. The clinical diagnosis includes a thorough history of the patient following the above principles, a history of carers (parents, partners, siblings etc.) and behavioral observations in a diagnostic process that usually takes several sessions. Psychometric tools like Australian Scale for Asperger’s Syndrome (ASAS) [37], Social Responsiveness Scale (SRS) [22], Bermond Vorst Alexithymia Questionnaire (BVAQ) [38], Adult Asperger Assessment (AAA, consisting of AQ [19] and EQ [20,39]), and Beck’s Depression Inventory (BDI) [40] are obtained in a routine procedure prior to clinical assessment and are used also for differential diagnostics. Additionally, instruments like Autism Diagnostic Interview – Revised (ADI-R) [41] and the Autism Diagnostic Observation Schedule (ADOS) [42,43] are applied in selected and unclear cases. In this study ADOS was performed in 7 patients of the AS-group, ADI-R was performed in 6 patients of the AS-group. The same is true for additional neuropsychological tests assessing executive and theory-of-mind capacities. The multiprofessional diagnostic team consists of three experienced senior consultant psychiatrists and two fully qualified senior psychologists. The final consensus diagnosis is made by all persons involved in the diagnostic process, which will invariably include at least two experienced consultant psychiatrists or psychologists.

### Ethical considerations

The study was carried out in accordance with the Code of Ethics of the Declaration of Helsinki and was approved by the Ethics Committee of the University of Freiburg, Germany (264/12).

## Results

### Sample characteristics

A total of 179 persons were included in our study between June 2012 and March 2013. The AS-group was composed of 57, the C-group of 56 and the PD-group of 66 patients (see Table 1). Age, gender distribution and educational level differed significantly – as expected – between groups. They were treated as independent factors and included in the calculations.

**Table 1:** **Sample characteristics**

	**Asperger’s syndrome- sample**	**Convenience sample**	**Psychiatric disorder-sample**	**p-value**
**N**	57	56	66	
**Demographic variables**				
**Gender**	**N (%)**	**N (%)**	**N (%)**	.001^1^
Male	36 (63.2)	21 (37.5)	21 (31.8)	
Female	21 (36.8)	35 (62.5)	45 (68.2)	
**Age**	**M (SD)**	**M (SD)**	**M (SD)**	<.001^2^
	40.2 (9.89)	30.4 (10.08)	36.4 (13.29)	
**Relationship status**	**N (%)**	**N (%)**	**N (%)**	.909^1^
Yes	27 (47.4)	28 (50.0)	34 (51.5)	
No	28 (49. 1)	26 (46.4)	30 (45.4)	
**Educational level**	**N (%)**	**N (%)**	**N (%)**	<.001^3^
Low	4 (7.0)	2 (3.6)	8 (12.1)	
Medium	12 (21.1)	1 (1.8)	28 (42.4)	
High	41 (71.9)	53 (94.6)	30 (45.5)	
**Clinical variables**				
**Psychological distress**	**M (SD)**	**M (SD)**	**M (SD)**	<.001^2^
SCL-K-9	1.17 (0.85)	0.66 (0.45)	1.58 (0.84)	
**Symptoms of personality disorder**	**M (SD)**	**M (SD)**	**M (SD)**	<.001^2^
SAPAS	4.63 (1.58)	1.61 (1.09)	4.05 (1.69)	

M = mean; SD = standard deviation, p-value based on ^1^chi-squared test, ^2^variance analysis, ^3^Fischer’s exact test.

The 66 patients with psychiatric disorders were composed of: 15 with depression (3 men, 12 women), 13 with schizophrenia (4 men, 9 women), 9 with ADHD (4 men, 5 women), 13 with obsessive-compulsive disorder (9 men, 4 women) and 16 with borderline personality disorder (1 man, 15 women). The average age of this group was 36.4 years.

Psychological distress (SCL-K-9) was significantly lower in the control group than in the AS-group and the PD-group (p < .001). The participants of the C-group showed significantly fewer symptoms of personality disorders than the participants of the AS- and the PD-group (p < .001).

### Reading level of the instrument

We calculated the reading level of the German version of the instrument using an online-software (fleschindex.de). Our text consists of 23 sentences, 123 words, 244 syllables and 873 characters. A Flesch-Reading-Ease-Score of 59 was calculated, indicating that the difficulty of the text is easy to moderate.

### Dimensionality of the questionnaire

Two factors showed a value of >1 in the explorative factor analysis. The graphic analysis of the Scree plot was in favor of a one-factor solution, however. Since only one item (item 6) loaded clearly on the second factor, the factor loading of the rotating factor analysis also pointed in favour of a one-factor solution. Thus, the explorative factor analysis was repeated excluding item 6. The second factor analysis resulted clearly in a one-factorial solution since only one factor retained a value >1. This factor had a value of 6.80 and explained 56.70% of the variance. Since one item lacked factor reliability > .6 (item 12: factor reliability: .567), it was also excluded and the analysis was repeated. This final model provided a single factor solution with the value of 6.51 and explained 59.21% of the variance. Factor reliabilities lay between .648 and .874 and only two items showed factor reliability under 7.

### Reliability

Reliability analysis of the whole scale showed a Cronbach’s α of .930. Corrected item-total correlation ranged from 596 to 831.

### Validity

**Figure 1:**
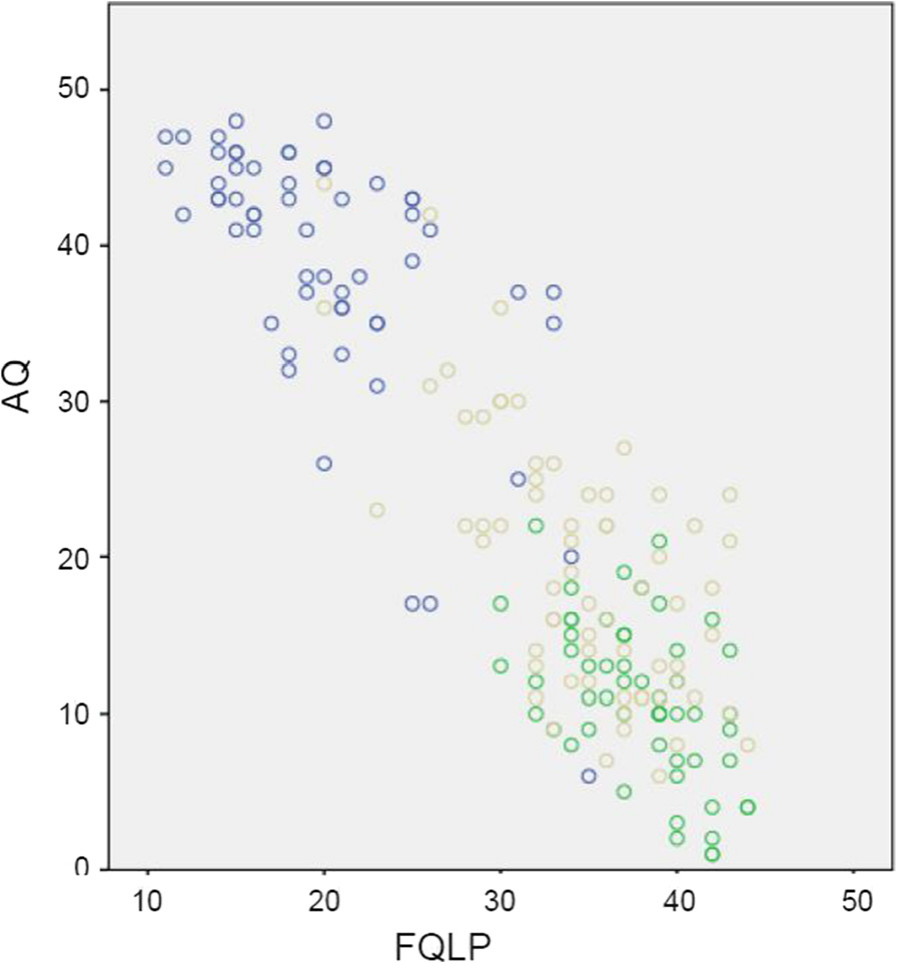
**Unadjusted AQ and FQLP scores of the AS-group, the C-group and the PD-group.** Legend: AS-group: blue circles; C-group: green circles; PD-group: yellow circles.

Convergent validity was investigated by analysing the correlation of the self-rated pragmatic speech abilities (FQLP) with two well-established questionnaires for ASD-symptoms (EQ and AQ) controlling for word pool (MWT-B), psychological distress (SCL-K-9), and symptoms of personality disorder (SAPAS). A correlation of r = .754 (p < .001) was found between FQLP and EQ. The correlation between FQLP and AQ was r = -.824 (inverse scoring of the scales, p < .001, Figure 1). Divergent validity was investigated by analysing the correlation of self-rated pragmatic speech abilities with symptoms of psychopathology, education and word pool. A moderate correlation of r = -.523 (inverse scoring of the scales, p < .001) was found between the self-rated pragmatic speech abilities (FQLP) and self-rated symptoms of personality disorders (SAPAS). The correlation between self-rated pragmatic speech abilities (FQLP) and self-rated psychological distress (SCL-K-9) was low (inverse scoring of the scales, r = −.237) but still significant (p = .001). A low (r = .215) but significant (p = .004) correlation (Spearman's rank correlation coefficient) between FQLP and education was found. No significant correlation was found between the self-rated pragmatic speech abilities (FQLP) and the word pool (MWT-B) (r = −.070, p = .361).

**Figure 2:**
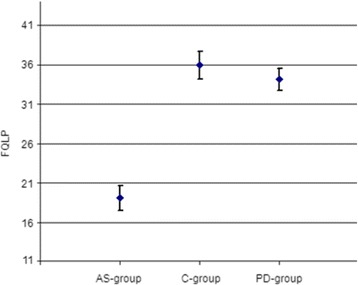
**Estimated marginal mean of the FQLP with 95% confidence interval.**

In a generalized linear model we analysed the effect of the variables *group*, *education*, *sex* and *age* on self-rated pragmatic speech abilities (FQLP). We found a significant constant term (p < .001), a significant group effect (p < .001), and a significant effect of the educational status on self-rated pragmatic speech abilities (FQLP). No significant effects were found for sex (p = .787) and age (p = .345). The estimated marginal mean of the FQLP was 19.06 (SE = .80) in the AS-group, 35.98 (SE = .88) in the C-group and 34.16 (SE = .69) in the PD-group (Figure 2). The estimated marginal means of all three groups differed significantly from the overall mean.

### Diagnostic accuracy

**Figure 3:**
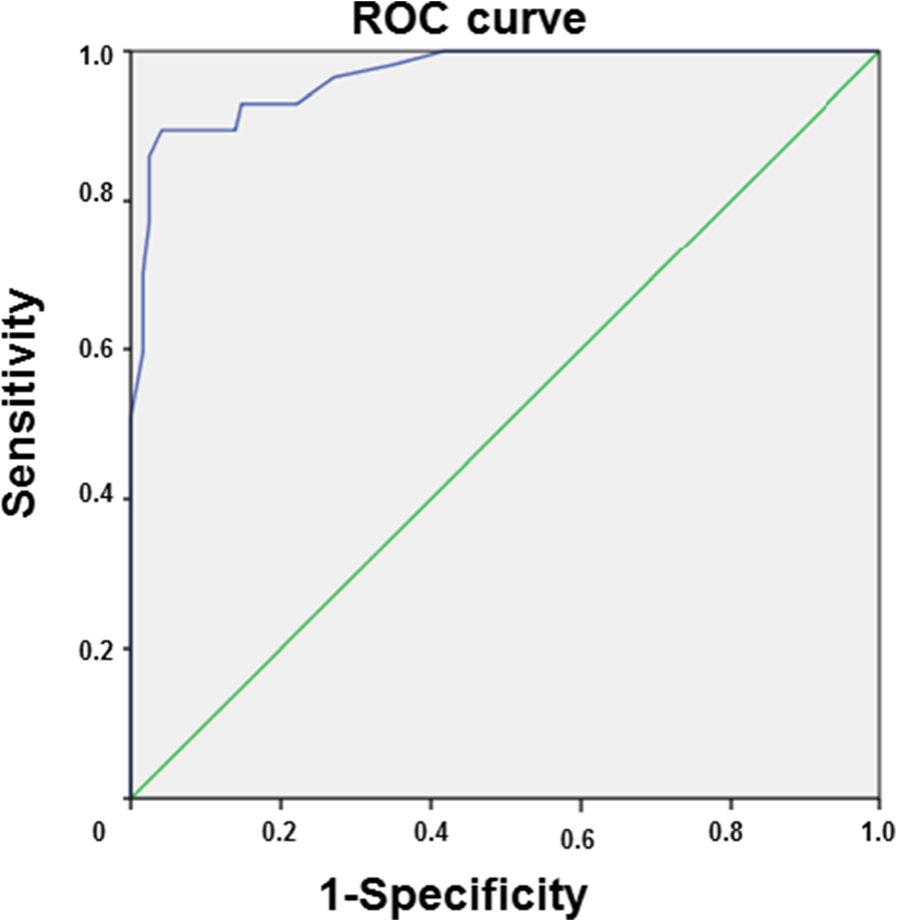
**ROC-Curve.**

The receiver operating characteristics curve showed an excellent diagnostic accuracy of the FQLP (see Figure 3). The area under the receiver characteristic curve was .97 (95% CI .94-.99). Sensitivity and specificity are depicted for different cut-off points in Table 2.

**Table 2:** **Diagnostic accuracy of the FQLP in identifying patients with AS**

**Questionnaire scores**	**Sensitivity in %**	**Specificity in %**
<31	90	86
<32	93	85
<33	93	78
<34	97	73
<35	98	65

## Discussion

Psychometric properties of the FQLP, a self-assessment questionnaire which focusses on difficulties with pragmatic speech, were explored in a sample of 57 AS patients, 66 patients with other psychiatric disorders, and a convenience sample of 56 people.

The FQLP requires a reading level typically found in people with medium education level. As 93% of the AS-group had a medium or high education level, the vast majority should have been able to read and understand our questionnaire without any difficulties.

No definite one-dimensional solution was found regarding the dimensionality of the first version of the questionnaire with 13 items. Furthermore, not all items showed sufficient factor reliability. Therefore, two items were removed. This procedure resulted in a scale with good psychometric parameters: The 11-item-FQLP is a one-dimensional construct with high internal consistency and a very high reliability, represented by a very high Cronbach’s α. Good convergent validity could also be demonstrated for the FQLP with AQ and EQ, which are well established questionnaires regarding symptoms of AS. FQLP, and AQ and EQ showed a very high statistical correlation even though they assess different aspects of the AS respectively. As hypothesized, the FQLP showed only moderate correlation with psychological distress and with symptoms of personality disorders. The correlation with education was significant, but low. Limited influence of education on self-rated pragmatic speech abilities was found. Self-perception of language comprehension measured with the FQLP is an independent construct that can clearly be distinguished from education. Discriminant validity was assessed in comparison to MWT-B, which describes vocabulary abilities, in order to clarify whether the FQLP only assesses basic linguistic (for example semantic) abilities. This resulted in no significant correlation, demonstrating that the FQLP captures a construct which is fundamentally different from vocabulary abilities. As far as criterion validity is concerned, we found significantly lower FQLP-scores in the AS-group compared to the C-group or the PD-group. On the basis of a receiver operating characteristics curve a very high diagnostic accuracy of the FQLP could be demonstrated: In comparison with the healthy population sample and the PD-group, the FQLP showed a high predictive value regarding AS.

Although the main results were surprisingly clear, there are some limitations to the study: First, because of the small sample size the result could not be cross-validated. Second, although no indication arose that age or sex had any influence on the FQLP score, it must be stated as a limitation that the groups differed in these respects. Third, the subgroups were too small to allow for a differentiated evaluation of the FQLP scores of the individual psychiatric illnesses. Therefore, reliable statements about their perception of their respective pragmatic peculiarities are not yet possible. It would make sense to particularly assess the group of schizophrenic patients in a larger group using the FQLP, since pragmatic peculiarities have repeatedly been described for this group [37]. Perhaps the FQLP questions relating to childhood and youth could be an especially helpful tool in distinguishing these two conditions. Fourth, it is difficult to judge a possible bias of the psychiatrists who diagnosed AS in the patients examined here: All patients were recruited by the Clinic of Psychiatry and Psychotherapy of the University Medical Center Freiburg, and all diagnoses were made there. Diagnoses are formed according to the ICD-10-Criteria. However, it cannot be excluded that the interest which the team has in the pragmatic peculiarities of AS patients has influenced the diagnostic process, leading perhaps to a higher percentage of patients receiving the diagnosis AS who demonstrated pragmatic language abnormalities. This may then have contributed to the fact that many AS patients reported pragmatic peculiarities in this study. According to our own judgement, this AS-sample-related selection bias has only a moderate effect if at all, because the diagnostic procedure was carried out very carefully and the questionnaires were completed independently by participants and patients without knowledge of the study hypothesis. Still a replication of our results by an independent work group is desirable. Fifth, in the fifth edition of the Diagnostic and Statistical Manual of Mental Disorders (DSM-5, 2013) the diagnosis of social (pragmatic) communication disorder (SCD) was newly established. Since SCD concerns a new diagnostic category, which also finds no correspondence in the ICD to date, no sufficient number of diagnosed patients is yet available who would qualify for participation in such a study. It must, however, be presumed that the FQLP as differential diagnostic instrument between AS and SCD will be less helpful, since it targets just that range of symptoms that both diseases have in common. In those cases, other instruments should be made use of. Nonetheless, this does not affect the demonstrated high sensitivity and specificity of our instrument with respect to AS in distinction to other psychiatric diseases. Where it is a clinical issue to distinguish between AS and obsessive compulsive disorder, for example, or between AS and ADHD, the FQLP can make a real contribution to the differential diagnosis. As mentioned above, care has been taken to strictly follow the NICE-guidelines in establishing a diagnosis, and pragmatic language abnormalities are not of high relevance in this process. Therefore, it is very unlikely that SCD-patients were falsely diagnosed as AS-patients.

Sixth, the questions of the FQLP implicitly require a high degree of education, since words such as “metaphor”, “ambiguity”, “rational analysis” or “intuitively” must be readily understood. The FQLP questions also demand quite a high level of self-reflection and introspection. Questions such as: “I use rational analysis to work out the meanings of metaphors” cannot be answered without introspective ability. Thus, the questionnaire might turn out to be suitable rather for high-functioning and well-educated individuals. Seventh, we did not define a cut-off point for the FQLP prior to our investigation. The cut-off criterion was retrospectively optimized and post-hoc set at the value of 32. This means that the results concerning specificity and sensitivity have to be interpreted cautiously, at least until the findings have been replicated in the context of further prospective studies. Eighth, the inclusion of subjects for the C-group using personal contacts of the authors might have introduced a bias towards high linguistic pragmatic abilities in this group. This might have led to a more significant difference between the C-group and the AS-group. Since the difference between the C-group and the PD-group – which was obviously not influenced by the mentioned bias – was comparatively low, we assume this effect to be rather small. Nineth, the nature of what the FQLP measures can be questioned. Gernsbacher and Pripas-Kapit [16] argue that the deficits in pragmatic language which are found in autism could also be a consequence of deficits in general language comprehension rather than of specific pragmatic impairments. If this were true, the FQLP would not evaluate pragmatic abilities, but language comprehension in general. This cannot be excluded and can be considered as a limitation of the FQLP. In particular this would imply that the name of our questionnaire may be misleading. Nevertheless this objection neither challenges the finding that the FQLP has been shown to measure a one-dimensional construct with a high internal consistency which is fundamentally different from vocabulary abilities, nor does it challenge the result that the FQLP showed a high diagnostic accuracy in differentiating AS from other psychiatric conditions.

Finally, there are a few more points which should be discussed: The one-dimensionality of the FQLP is striking in light of the fact that the items of the FQLP relate to rather different aspects of language comprehension – at least at first glance: They investigate the understanding of irony and metaphors, the strategies to decipher figurative language, the participants’ principal ideas how language should be (“In an ideal language, there would be no ambiguity of meaning”) and the extent to which participants feel they understand other persons' utterances. In our opinion, the one-dimensionality indicates that impairments in the various aspects of language comprehension assessed by the FQLP can be traced to a single underlying syndrome in the case of persons with AS.

Another interesting observation is the high convergent validity of AQ/EQ and FQLP. The results indicate that pragmatic speech abilities and the symptom ratings are highly related constructs or may even assess different aspects of the same construct. This in turn would mean that the peculiarities of pragmatic language investigated by the FQLP are a symptom of AS.

The moderate correlation between general psychopathology or symptoms of personality disorders and the FQLP results may show that pragmatic peculiarities are found also in general psychopathology and in personality disorders. However, since the non-autistic patient group, which had high scores in general psychopathology, showed only mild abnormalities in the FQLP, we assume that the constructs “psychopathology”, “personality disorders” and “pragmatic abnormalities” exist primarily independently of each other and that this moderate correlation can be explained by the frequent comorbidities of autism spectrum disorders [34].

Since no correlation resulted between the results of the FQLP and the MWT-B, pragmatic peculiarities explored by the FQLP are probably independent of vocabulary skills. One could object that tests of vocabulary like the MWT-B do not allow conclusions with respect to language comprehension [44]. For this reason, it cannot be excluded that the FQLP measures general language comprehension – and not specific pragmatic abilities. Nevertheless, it is remarkable that both the educational level and the FQLP-score of the AS-group were significantly higher than that of the PD-group. The high educational level in the AS-group argues against significant problems of general language comprehension. This indicates that problems of general language comprehension are probably not a proper explanation for the low FQLP-score in the AS-group.

The high percentage of women in our group of patients diagnosed with AS is remarkable. Older surveys assume a male to female ratio of 8:1 [45]. However, surveys focussing only on adults find more often a higher percentage of women (2:1, 3:1) [34,35]. Lehnhardt, who found a male to female ratio of 2:1, comes up with some plausible explanations for this phenomenon [35].

The most substantial finding of this study is the remarkably high predictability of the FQLP regarding AS. As our control group consisted of people without mental disorder and patients with different psychiatric disorders this result can be seen as a clear indication that the construct examined by the FQLP is quite specific to the peculiarities of AS. Furthermore, the results of the correlational analyses of the divergent validity indicate that the FQLP measures a construct that can clearly be distinguished from general psychological distress or specificities of personality. Comparing the FQLP to other questionnaires for AS, it shows comparable or even better results: The short version of the AQ, which has 10 items, shows – depending on the cut-off-point – specificity between 73% and 89%, and sensitivity between 71% and 92% [46]. Nota bene in the aforementioned study the AS-patients were compared to a control group of healthy subjects. Even the “long” AQ with 50 items used in the screening of AS exhibited a sensitivity of 95% and a specificity of only 52% [47]. This study examined people who were already suspect of AS before the study, which explains the low specificity. The EQ demonstrated a sensitivity of 81% and a specificity of 88% [20]. The Social Communication Questionnaire (SCQ) [23] was evaluated quite well, showing a sensitivity of 89% and a specificity of 91% [22] in a quite heterogeneous study sample. Since the SCQ is a 40-item questionnaire for the parents of autistic children, it is not totally comparable to the self-assessment questionnaires for adults like AQ, EQ or FQLP. At any rate our results show that – using a retrospectively optimized cutoff criterion – sensitivity (93% at a cutting-point of 32) and specificity (85% at a cutting-point at 32) of the FQLP are well comparable to already established questionnaires used for the screening of AS. Of course, it is by no means to be expected from a short questionnaire like the FQLP that it could replace proven diagnostic instruments, let alone the clinical diagnosis. In addition, its specificity relative to social communication disorder, which was not examined, is presumably only moderate. However, since the FQLP shows a good sensitivity and specificity relative to other psychiatric illnesses while being very short (a mere 11 questions), we assume, on the basis of the results shown here, that it is very suitable as a diagnostic instrument for AS in psychiatric practice and may complement the conventional diagnostic process well.

## Conclusion

The FQLP is a reliable, brief and valid instrument. First results regarding sensitivity and specificity are highly promising.

### Future directions

The aim of future research could be to have the psychometric properties of the FQLP studied by a group of independent researchers. Further, it would be interesting to know which scores patients with SCD attain and to what extent FQLP can be used as diagnostic instrument for SCD.

## Additional files

Additional file 1:
**Freiburg Questionnaire of linguistic pragmatics.**
Additional file 2:
**Freiburger Fragebogen zur Sprachpragmatik.**
