# Supplementary material for: Freiburg Questionnaire of linguistic pragmatics (FQLP): psychometric properties based on a psychiatric sample
Source: BMC Psychiatry. 2014 Dec 24;14:374. doi: 10.1186/s12888-014-0374-9 (PMC4296675; doi:10.1186/s12888-014-0374-9)
Supplement: Additional file 1: — Freiburg Questionnaire of linguistic pragmatics. [file 12888_2014_374_MOESM1_ESM.docx]

| Name: | ___________________ | |  |
| --- | --- | --- | --- |
| Date: | ___________________ | |  |
| Birthday: | ___________________ | |  |
| Gender: | ❑ female | ❑ male | |
| Graduation: | ___________________ | |  |
| Education: | ___________________ | |  |
| First language: | ___________________ | |  |

**Freiburg Questionnaire of linguistic pragmatics**

Please read the following statements carefully and decide to what extend they apply to you. You have the following opportunities:

| *I agree* | *I tend to agree* | *I tend not to agree* | *I do not agree* |
| --- | --- | --- | --- |
| **🔾** | **🔾** | **🔾** | **🔾** |

Please mark one oft he opportunities with a cross, respectively, and don´t leave out one of the statements. In case of doubt please chose the opportunity which fits the best.

|  |  | *I agree* | *I tend to agree* | *I tend not to agree* | *I do not agree* |
| --- | --- | --- | --- | --- | --- |
| 1. | My comprehension of language differs from that of other people | **🔾** | **🔾** | **🔾** | **🔾** |
| 2. | I often don’t understand what other people are saying to me | **🔾** | **🔾** | **🔾** | **🔾** |
| 3. | In conversation, I find metaphors and/or sayings irritating | **🔾** | **🔾** | **🔾** | **🔾** |
| 4. | I intuitively comprehend metaphors and/or sayings I have never heard before | **🔾** | **🔾** | **🔾** | **🔾** |
| 5. | I consider metaphors and/or sayings to be unnecessary | **🔾** | **🔾** | **🔾** | **🔾** |
| 6. | I recognise expressions which are not meant literally due to the fact that I have heard them in the past and I misunderstood them at that time | **🔾** | **🔾** | **🔾** | **🔾** |
| 7. | I usually recognise irony easily | **🔾** | **🔾** | **🔾** | **🔾** |
| 8. | At school, I often misunderstood what my teachers and classmates said to me | **🔾** | **🔾** | **🔾** | **🔾** |
| 9. | I have made a conscious effort to improve my comprehension of metaphors/sayings | **🔾** | **🔾** | **🔾** | **🔾** |
| 10. | I use rational analysis to work out the meanings of metaphors and/or sayings | **🔾** | **🔾** | **🔾** | **🔾** |
| 11. | In an ideal language, there would be no ambiguity of meaning | **🔾** | **🔾** | **🔾** | **🔾** |
|  |  |  |  |  |  |
