# Supplementary material for: Freiburg Questionnaire of linguistic pragmatics (FQLP): psychometric properties based on a psychiatric sample
Source: BMC Psychiatry. 2014 Dec 24;14:374. doi: 10.1186/s12888-014-0374-9 (PMC4296675; doi:10.1186/s12888-014-0374-9)
Supplement: Additional file 2: — Freiburger Fragebogen zur Sprachpragmatik. [file 12888_2014_374_MOESM2_ESM.docx]

| Nach- und Vorname: | ___________________ | |  |
| --- | --- | --- | --- |
| Das heutige Datum: | ___________________ | |  |
| Geboren am: | ___________________ | |  |
| Geschlecht: | ❑ weiblich | ❑ männlich | |
| Schulabschluss: | ___________________ | |  |
| Hochschulabschluss/Berufsausbildung: | ___________________ | |  |
| Muttersprache: | ___________________ | |  |

**Freiburger Fragebogen zur Sprachpragmatik - Kurzversion**

Im Folgenden finden Sie einige Aussagen über verschiedene Aspekte von Sprache. Bitte lesen Sie sich die Aussagen durch und entscheiden Sie inwieweit die jeweilige Aussage für Sie zutrifft. Sie haben folgende Antwortmöglichkeiten zur Auswahl:

| *ich stimme zu* | *ich stimme eher zu* | *ich stimme eher nicht zu* | *ich stimme nicht zu* |
| --- | --- | --- | --- |
| **🔾** | **🔾** | **🔾** | **🔾** |

Bitte machen Sie je ein Kreuz in das jeweilige Antwortkästchen. Lassen Sie bitte keine Aussage aus und kreuzen Sie im Zweifelsfall die Antwortmöglichkeit an, die noch am ehesten auf Sie zutrifft.

*Zum Beispiel:*

| „Mein Sprachverständnis unterscheidet sich von dem anderer Menschen“ | **🔾** | **🔾** | **🔾** | **🔾** |
| --- | --- | --- | --- | --- |

In diesem Fall ist das Kästchen mit der Ausprägung „Ich stimme eher nicht zu“ angekreuzt, was bedeutet, dass sich Ihr Sprachverständnis Ihrer Einschätzung nach eher nicht von dem anderer Menschen unterscheidet.

|  |  | *ich stimme zu* | *ich stimme eher zu* | *ich stimme eher nicht zu* | *ich stimme nicht zu* |
| --- | --- | --- | --- | --- | --- |
| 1. | Mein Sprachverständnis unterscheidet sich von dem anderer Menschen | **🔾** | **🔾** | **🔾** | **🔾** |
| 2. | Ich verstehe oft nicht, was andere mir sagen wollen | **🔾** | **🔾** | **🔾** | **🔾** |
| 3. | Im Gespräch finde ich Metaphern und/oder Sprichwörter irritierend | **🔾** | **🔾** | **🔾** | **🔾** |
| 4. | Mir nicht bekannte Metaphern und/oder Sprichwörter erschließen sich mir intuitiv | **🔾** | **🔾** | **🔾** | **🔾** |
| 5. | Metaphern und/oder Sprichwörter halte ich für unnötig | **🔾** | **🔾** | **🔾** | **🔾** |
| 6. | Ich erkenne nicht-wörtlich gemeinte Ausdrücke daran, dass ich sie in der Vergangenheit schon einmal gehört und damals missverstanden habe | **🔾** | **🔾** | **🔾** | **🔾** |
| 7. | Ironie erkenne ich meistens ohne Probleme | **🔾** | **🔾** | **🔾** | **🔾** |
| 8. | In der Schulzeit habe ich Aussagen meiner Lehrer und Mitschüler häufig missverstanden | **🔾** | **🔾** | **🔾** | **🔾** |
| 9. | Ich habe bewusst daran gearbeitet, Metaphern/Sprichwörter besser zu verstehen | **🔾** | **🔾** | **🔾** | **🔾** |
| 10. | Ich erschließe mir die Bedeutungen von Metaphern etc. durch rationales Analysieren | **🔾** | **🔾** | **🔾** | **🔾** |
| 11. | In einer idealen Sprache gibt es keine Zwei- oder Mehrdeutigkeiten | **🔾** | **🔾** | **🔾** | **🔾** |
|  |  |  |  |  |  |
